# Long-term LVEF trajectories in patients with type 2 diabetes and heart failure: diabetic cardiomyopathy may underlie functional decline

**DOI:** 10.1186/s12933-020-01011-w

**Published:** 2020-03-23

**Authors:** María Teresa Julián, Núria Alonso, Josep Lupón, Giovana Gavidia-Bovadilla, Elena Ferrer, Marta de Antonio, Jorge López-Ayerbe, Mar Domingo, Evelyn Santiago-Vacas, Elisabet Zamora, Pau Codina, Pedro Moliner, Julio Núñez, Javier Santesmases, Manel Puig-Domingo, Antoni Bayes-Genis

**Affiliations:** 1 Heart Failure Unit, Hospital Universitari Germans Trias i Pujol, Badalona, Barcelona, Spain; 2 Endocrinology Department, Hospital Universitari Germans Trias i Pujol, Badalona, Barcelona, Spain; 3 Centre of Biomedical Research on Diabetes and Associated Metabolic Diseases (CIBERDEM), Instituto de Salud Carlos III, Madrid, Spain; 4 Cardiology Department, Hospital Universitari Germans Trias i Pujol, Badalona, Barcelona, Spain; 5 Department of Medicine, Universitat Autònoma de Barcelona, Barcelona, Spain; 6 CIBER Cardiovascular, Instituto de Salud Carlos III, Madrid, Spain; 7 Department of e-Health, Eurecat, Technological Center of Catalonia, Barcelona, Spain; 8 Cardiology Department, Hospital Clínico Universitario, INCLIVA, Valencia, Spain; 9 Department of Medicine, Universitat de València, Valencia, Spain

**Keywords:** Heart failure, Diabetes mellitus, Ejection fraction, Etiology, Long-term, Follow-up

## Abstract

**Background:**

Left ventricular ejection fraction (LVEF) trajectories and functional recovery with current heart failure (HF) management is increasingly recognized. Type 2 diabetes mellitus (T2D) leads to a worse prognosis in HF patients. However, it is unknown whether T2D interferes with LVEF trajectories. The aim of this study was to prospectively assess very long-term (up to 15 years) LVEF trajectories in patients with and without T2D and underlying HF.

**Methods:**

Ambulatory patients admitted to a multidisciplinary HF clinic were prospectively evaluated by scheduled two-dimensional echocardiography at baseline, 1 year, and then every 2 years afterwards, up to 15 years. Statistical analyses of LVEF change with time were performed using the linear mixed effects (LME) models, and locally weighted error sum of squares (Loess) curves were plotted.

**Results:**

Of the 1921 patients, 461 diabetic and 699 non-diabetic patients with LVEF < 50% were included in the study. The mean number of echocardiography measurements performed in diabetic patients was 3.3 ± 1.6. Early LVEF recovery was similar in diabetic and non-diabetic patients, but Loess curves showed a more pronounced inverted U shape in diabetics with a more pronounced decline after 9 years. LME analysis showed a statistical interaction between T2D and LVEF trajectory over time (p = 0.009), which was statistically significant in patients with ischemic etiologies (p < 0.001). Other variables that showed an interaction between LVEF trajectories and T2D were male sex (p = 0.04) and HF duration (p = 0.008).

**Conclusions:**

LVEF trajectories in T2D patients with depressed systolic function showed a pronounced inverted U shape with a marked decline after 9 years. Diabetic cardiomyopathy may underlie the functional decline observed.

## Background

Heart failure (HF) is a multidimensional syndrome with a wide variety of symptoms and signs associated with cardiac functional and structural abnormalities, and carries a high morbidity and mortality [1, 2]. Patients with type 2 diabetes (T2D) have a two- to fivefold increased risk of developing HF compared to non-diabetic patients, and 15–25% of patients with HF have diabetes. This risk of HF remains high despite achieving control of other major cardiovascular risk factors [3–5]. Moreover, the presence of T2D contributes to worse outcomes in HF. Patients with T2D are more likely to be hospitalized for HF, to be re-admitted for HF, and have a higher risk of cardiovascular and all-cause mortality than those without diabetes and HF [6, 7]. Insulin resistance and hyperglycemia, the key central metabolic disturbances in T2D, lead to specific structural and metabolic abnormalities that adversely affect myocardial function [8].

Advances in HF treatment including drug therapy, devices, coronary revascularization, and valvular repair are responsible for systolic function improvement in patients with depressed left ventricular ejection fraction (LVEF) [3, 9], which might be maintained for a decade. We recently described the dynamic long-term trajectory of LVEF in a real-life cohort of HF patients with depressed systolic function of diverse etiology. Data from this study revealed that LVEF trajectory is highly variable dependent upon etiology, HF duration, and sex, but in general showed a significant improvement at 1 year, with an LVEF rise up to decade and a slow decline thereafter [10]. It remains unclear whether the presence of T2D interferes with the improvement and trajectories of LVEF.

Accordingly, the aim of the present study was to assess very long-term (up to 15 years) LVEF trajectories in T2D HF patients (from ischemic and non-ischemic etiologies), and to compare these trajectories with those observed in non-diabetic patients.

## Methods

### Study design and population

This study is a T2D-focused post hoc sub analysis of a previously reported cohort [10]. All consecutive ambulatory patients referred to a structured multidisciplinary HF clinic of a university hospital between August 2001 and December 2015, regardless of etiology, were considered for the study. During the 15-year period, clinical pathways and referral geographical area, encompassing ~ 850,000 inhabitants in the northern Barcelona Metro Area, remained stable. The criteria for referral to the HF clinic were HF with at least one hospitalization and/or depressed systolic function [10, 11]. Patients were treated according to a unified protocol. During the baseline visit, patients provided written consent for the use of their clinical data for research purposes.

### Inclusion criteria

The main inclusion criteria for this study were having had at least two echocardiography measurements, one at baseline and the other during follow-up. Patients undergoing heart transplantation or cardiac resynchronization therapy after the second echocardiography were censored at the time of the intervention. The study was performed in compliance with the law protecting personal data in accordance with the international guidelines on clinical investigation of the World Medical Association’s Declaration of Helsinki.

### Study outcome and follow-up

The primary outcome of the study was prospectively assess very long-term (up to 15 years) LVEF trajectories in patients with and without T2D and underlying HF. All patients were seen regularly during follow-up visits at the HF clinic according to their clinical needs and treated according to a unified protocol [10, 11]. Follow-up visits included a minimum of 1 visit with a nurse every 3 months and 1 visit with a physician (cardiologist, internist, or family physician) every 6 months, as well as optional visits with specialists in geriatrics, psychiatry, and rehabilitation. During the baseline visit, patients provided written consent for the use of their clinical data for research purposes [10, 11].

### Echocardiogram studies

LVEF was prospectively evaluated at baseline and at 1, 3, 5, 7, 9, 11, 13, and 15 years of follow-up using two-dimensional echocardiography by image expert cardiologists [10]. LVEF was obtained from apical two- and four-chamber views and was calculated using the Simpson’s method. All echocardiograms were revised for accuracy by expert staff.

### T2D diagnosis

A diagnosis of T2D was made when at least one of the following criteria was met: (1) a diagnosis of T2D was previously established and recorded in the patient’s electronic History; (2) fasting plasma glucose ≥ 126 mg/dL or hemoglobin A1C ≥ 6.5% identified by laboratory testing [12]; or (3) had a current prescription for oral hypoglycemic medication or insulin.

### Statistical analysis

Categorical variables were expressed as absolute numbers and percentages. Continuous variables were expressed as the mean (standard deviation) or median (quartiles Q1 to Q3) according to normal or non-normal distributions. Normal distribution was assessed with a normal Q–Q plot. Locally weighted error sum of squares (Loess) curves were plotted for diabetic and non-diabetic patients and for pre-specified study subgroups. Loess regression is a non-parametric approach developed in 1988 [13]. Loess curves are useful for observing a trend or relationship for non-linear data observed over time. Loess moves along the dataset looking at chunks at a time, fitting a set of local regression lines computed on observed data (missing values are omitted) and connecting these lines to make a smooth line. Missing data due to loss to follow-up was assumed to be at random as there was no evidence that not attending the scheduled visit had anything to do with LVEF. Locally weighted regression is based on a weight function, which gives the greatest weight to observations that are closest to the focal observation. Statistical analyses of LVEF change over time were performed using the linear mixed effects (LME) model, which takes into account the group level structure in the data by simultaneous assessing effects within and across groups. LME models incorporate both fixed effects and random effects [14] and describe the relationship between a response and covariates that have been observed along with the response [15]. In this study, LME models were developed to evaluate and compare the effect of time on LVEF change for diabetic and non-diabetic patients and for pre-specified subgroups according to HF etiology, HF duration at baseline and sex. We hypothesized that there are important individual-level effects and that patients have similar rates of change over time. Thus, we fitted the “Random Intercept LME models,” where the measured value of LVEF is assumed to have a set of parameters fixed across individuals, including a specific random effect per individual. Because the form of the Loess curves suggested at least a quadratic in time, all LME models included both the linear term time and the quadratic time ‘timeˆ2’ as fixed effects. By adding the quadratic term ‘timeˆ2’ to the models, we evaluated whether the effect of time significantly changed over time. The Wald Chi-squared test was applied to evaluate the accuracy of the estimates of the effect of time on LVEF values. Comparisons of LVEF between groups were also performed at every timepoint of the study using the paired *t*-test. Comparisons between included and excluded patients and between alive and dead patients were performed using the Student’s *t*-test, Mann–Whitney U test, or Chi-squared test as appropriate. Multivariable Cox regression analyses were performed with all-cause death and the composite all-cause death or HF hospitalization as the dependent variables and age, sex, diabetes mellitus, baseline LVEF, ischemic etiology and NYHA functional class as covariates. Recurrent HF-related hospitalizations were analyzed as crude incidence rates (expressed as number of hospitalizations per 100 patient-years) and by multivariable binomial negative regression, expressed as incidence rate ratios (IRRs). Only for the later analyses, out-of-hospital death due to HF was considered as an additional event. In a sensitivity analysis the influence of diabetic control-based on 2662 measurements of HbA1c—on LVEF trajectory and outcomes was also performed within diabetic patients. Optimal glycemic control was considered when median values of HbA1c for each patient were ≤ 7.5%.

Statistical analyses were performed using SPSS 24 (SPSS, Inc., Chicago, IL, USA) and R (A Language and Environment for Statistical Computing) by the R Core Team (2017; R Foundation for Statistical Computing, Vienna, Austria). For LME models, we used the nlme R package (version 3.1-131.1). Two-sided p < 0.05 was considered statistically significant. The data, analytic methods, and study materials will not be made available to other researchers for the purposes of reproducing the results or replicating the procedure. J.L. had full access to all data in the study and takes responsibility for its integrity and the data analysis.

## Results

A total of 1921 patients were admitted to the HF clinic from August 2001 to December 2015, and 1656 had an LVEF < 50%. After the exclusion criteria were applied, 1160 patients had a minimum of two LVEF measurements and comprised the study population [10]. Focusing on T2D patients, a range of 2 to 9 echocardiograms were performed per patient, in a total of 1534 patients as shown in Additional file 1: Figure S1. The mean number of echocardiograms performed in T2D patients (3.3 ± 1.6) was less than that performed in non-diabetics (3.8 ± 1.8; p < 0.001) and follow-up was also significantly shorter in T2D patients (5.8 ± 3.7 vs. 7 ± 4.1 years; p < 0.001).

Table 1 shows the clinical, biochemical, and echocardiographic characteristics of the studied cohort and treatment during follow-up based on the presence or absence of T2D. The main etiology in diabetic patients was ischemic heart disease (64.6%) followed by dilated cardiomyopathy (12.1%). Medical treatment was optimized according to international guidelines. Although diabetic patients received less angiotensin-converting enzyme inhibitor/angiotensin II receptor blockers treatment; in contrast they received more mineralocorticoid receptor antagonist, loop diuretic, digoxin, and ivabradine treatments. Comparison between included and not included patients due to lack of a second echocardiogram to address group bias, has been reported elsewhere [10]. No statistical differences were found in the variables mostly associated with LVEF dynamics such as sex, etiology, baseline LVEF, and HF duration. There was also no statistically significant difference in the prevalence of diabetes: included 461 (40%), excluded 118 (43.7%) (p = 0.23).

**Table 1:** Demographic, clinical, and therapeutic characteristics of patients at baseline and treatments during follow-up

	Diabetics	Non-diabetics	p-value	N
	N = 461	N = 699		
Age, years	66.4 ± 10.5	64 ± 13.2	0.001	1160
Male	338 (73.3)	549 (78.5)	0.04	1160
White	459 (99.6)	692 (99.0)	0.20	1160
Etiology			< 0.001	1160
Ischaemic heart disease	298 (64.6)	361 (51.6)		
Dilated cardiomyopathy	56 (12.1)	105 (15.0)		
Hypertensive	33 (7.2)	48 (6.9)		
Alcohol	16 (3.5)	52 (7.4)		
Drugs	7 (1.5)	27 (3.9)		
Valvular	23 (5.0)	49 (7.0)		
Other	28 (6.1)	57 (8.2)		
Previous AMI	238 (51.6)	306 (43.8)	0.009	
HF duration, months	7 (2–39)	6 (1–41)	0.10	1160
NYHA class			0.001	1160
I	14 (3.0)	46 (6.6)		
II	318 (60.0)	499 (71.4)		
III	125 (27.1)	152 (21.7)		
IV	4 (0.9)	2 (0.3)		
LVEF, %	30.4 ± 8.4	30.3 ± 8.6	0.96	1160
LVEDD, mm	60.6 ± 7.9	61.8 ± 8.6	0.02	1043
LVESD, mm	48.4 ± 9.4	49.8 ± 9.6	0.02	1027
Hypertension	333 (72.2)	380 (54.4)	< 0.001	1160
Anemia^a^	232 (52.3)	248 (36.3)	< 0.001	1127
Renal insufficiency^b^	239 (52.2)	249 (36.1)	< 0.001	1148
Atrial fibrillation/flutter	80 (17.4)	123 (17.6)	0.92	1160
LBBB	60 (13.0)	95 (13.6)	0.78	1160
Heart rate, bpm	72.2 ± 13.6	69.6 ± 15.1	0.003	1160
Blood pressure, mmHg	126.5 ± 21.9	124.8 ± 21.6	0.19	1160
BMI, kg/m^2^	27.7 (25–31.2)	26.5 (23.9–29.7)	< 0.001	1156
NTproBNP, ng/L	1825 (808–4363)	1530 (611–3344)	0.01	714
HF treatments (F-U), n (%)				
ACEI or ARB	421 (91.3)	663 (94.8)	0.02	1160
Beta-blocker	436 (94.6)	658 (94.1)	0.75	1160
MRA	333 (72.2)	445 (63.7)	0.002	1160
Loop diuretic	441 (95.7)	621 (88.8)	< 0.001	1160
Digoxin	213 (46.2)	264 (37.8)	0.004	1160
Ivabradine	113 (24.5)	122 (17.5)	0.003	1160
Sacubitril/valsartan	14 (3.0)	30 (4.3)	0.27	1160
CRT	23 (5.0)	45 (6.4)	0.30	1160
ICD	62 (13.4)	109 (15.6)	0.31	1160
Antidiabetic treatments				
Oral drugs baseline	248 (53.8)			461
Insulin baseline	175 (38.0)			461
Oral drugs F-U	367 (79.6)			461
Insulin F-U	305 (66.2)			461

Data in mean ± SD, median (IQR) or n (%)

*ACEI* angiotensin converting enzyme inhibitor, *ARB* angiotensin II receptor blocker, *BMI* body mass index, *CRT* cardiac resynchronization therapy, *eGRF* estimated glomerular renal filtration (CKD-EPI equation), *F-U* follow-up, *HF* heart failure, *ICD* implantable cardiac defibrillator, *LBBB* left bundle branch block, *LVEF* left ventricular ejection fraction, *LVEDD* left ventricular end-diastolic diameter, *LVESD* left ventricular end-systolic diameter, *MRA* mineralocorticoid receptor antagonist, *NYHA* New York Heart Association, *NTproBNP* N-terminal pro-brain natriuretic peptide

^a^According to W.H.O. criteria (< 13 g/dL in men and < 12 g/dL in women)

^b^eGFR (CKD-EPI equation) < 60 ml/min/1.73 m^2^

**Fig. 1:**
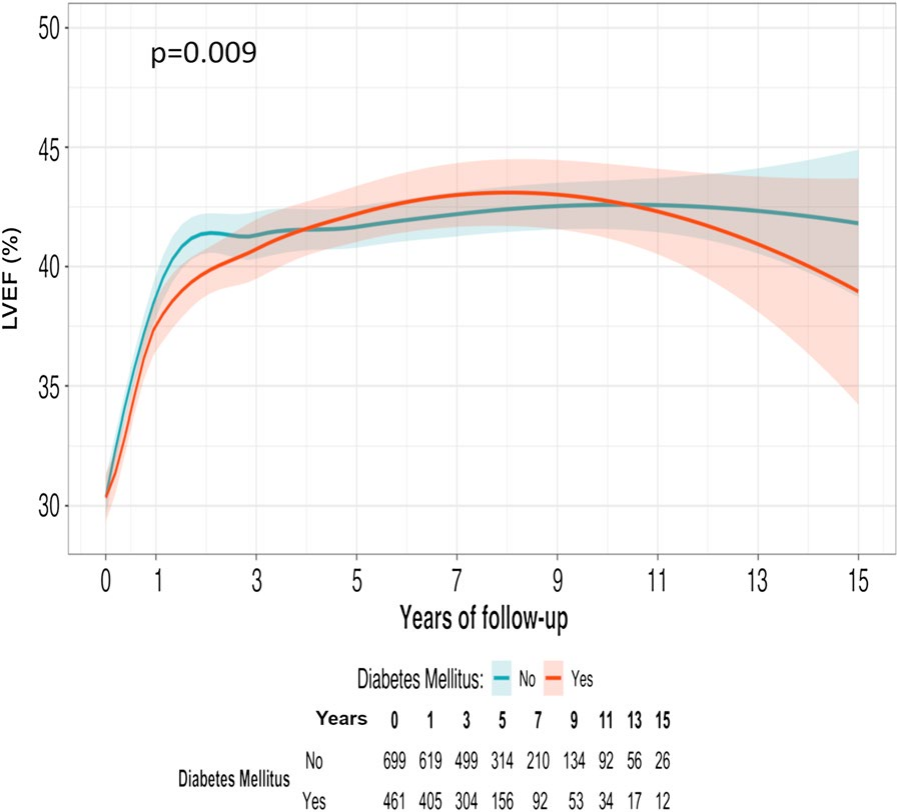
Loess spline curves of long-term LVEF trajectories. LVEF trajectory comparison between diabetic (orange) vs. non-diabetic (blue) patients. P for trajectory changes on LVEF < 0.001 for both groups. P for comparison between groups (interaction between trajectory changes and diabetes) = 0.009. Shaded regions displayed around curves represent the confidence interval at level = 0.95

Baseline characteristics of the study population stratified by HF etiology and diabetic status are presented in Additional file 2: Table S1. Co-morbidities such as anemia hypertension, and renal insufficiency were more prevalent in diabetic patients, both from ischemic and non-ischemic etiologies. Differences between diabetic and non-diabetic patients were generally similar in ischemic and non-ischemic etiologies, except regarding the use of loop diuretics and cardiac devices. Mean LVEF values in diabetic patients were 30.4% ± 8.4 (n = 461), 37.9% ± 11.6 (n = 417), 40.7% ± 12.3 (n = 304), 41.9% ± 12.4 (n = 156), 43% ± 13.2 (n = 92), 42.9% ± 12.4 (n = 53), 42% ± 11.2 (n = 34), 39.2% ± 11 (n = 17), and 40.6% ± 11.7 (n = 12) at baseline and 1, 3, 5, 7, 9, 11, 13, and 15 years, respectively. Dynamic trajectories of LVEF based on the presence or absence of T2D are illustrated in Fig. 1. Paired data comparisons showed statistical differences between baseline and 1 year (*p* < 0.001), and between 1 and 3 years (p < 0.001; Additional file 2: Table S2). As a whole, Loess splines of long-term LVEF trajectories in diabetic patients showed a more pronounced inverted U shape with a marked rise in LVEF during the first year (slightly lower than that of non-diabetics), followed by a more pronounced and earlier LVEF decline (p for trajectory < 0.001 for both groups; p for interaction between LVEF trajectory and diabetes = 0.009).

**Fig. 2:**
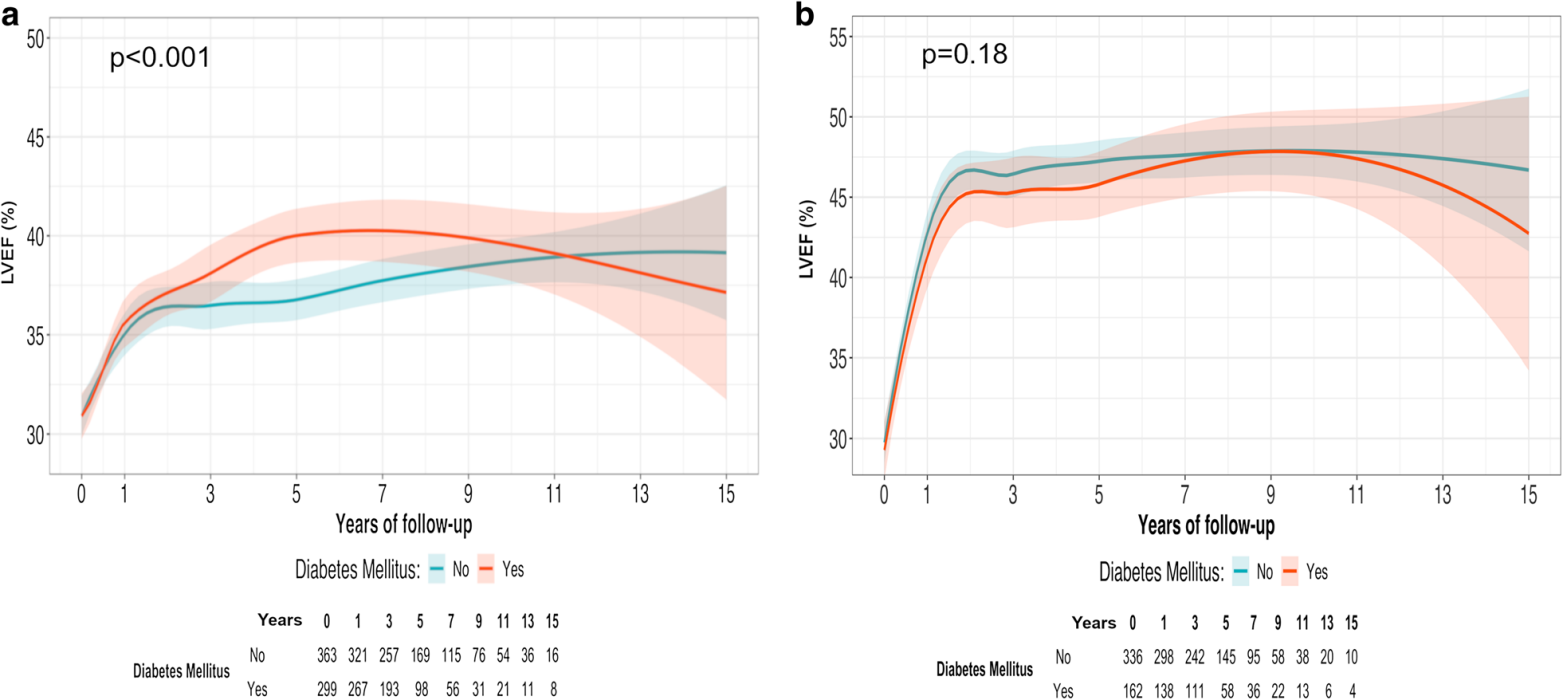
Loess spline curves of long-term LVEF trajectories based on etiology. **a** Patients from ischemic aetiology of HF; diabetic (orange) vs. non-diabetic (blue) patients. P value for trajectory changes on LVEF < 0.001 for both groups. P for comparison between groups (interaction between trajectory changes and diabetes) < 0.001. Shaded regions displayed around curves represent the confidence interval at level = 0.95. **b** Patients from non-ischemic aetiologies. P value for trajectory changes on LVEF < 0.001 for both groups. P for comparison between groups (interaction between trajectory changes and diabetes) = 0.18. Shaded regions displayed around curves represent the confidence interval at level = 0.95

Figure 2a, b shows LVEF trajectories relative to the etiology of HF, ischemia versus non-ischemia. Long-term LVEF decline was more apparent in T2D ischemic patients with HF (p for interaction < 0.001) (Fig. 2a). An interaction between T2D and LVEF trajectory in patients with longer HF duration (p = 0.008; Additional file 3: Figure S2A; Additional file 4: Figure S2B) and in males (p = 0.04; Additional file 5: Figure S3A) was also observed. In T2D women, no differences were observed in the dynamic trajectories of LVEF (Additional file 6: Figure S3B).

**Fig. 3:**
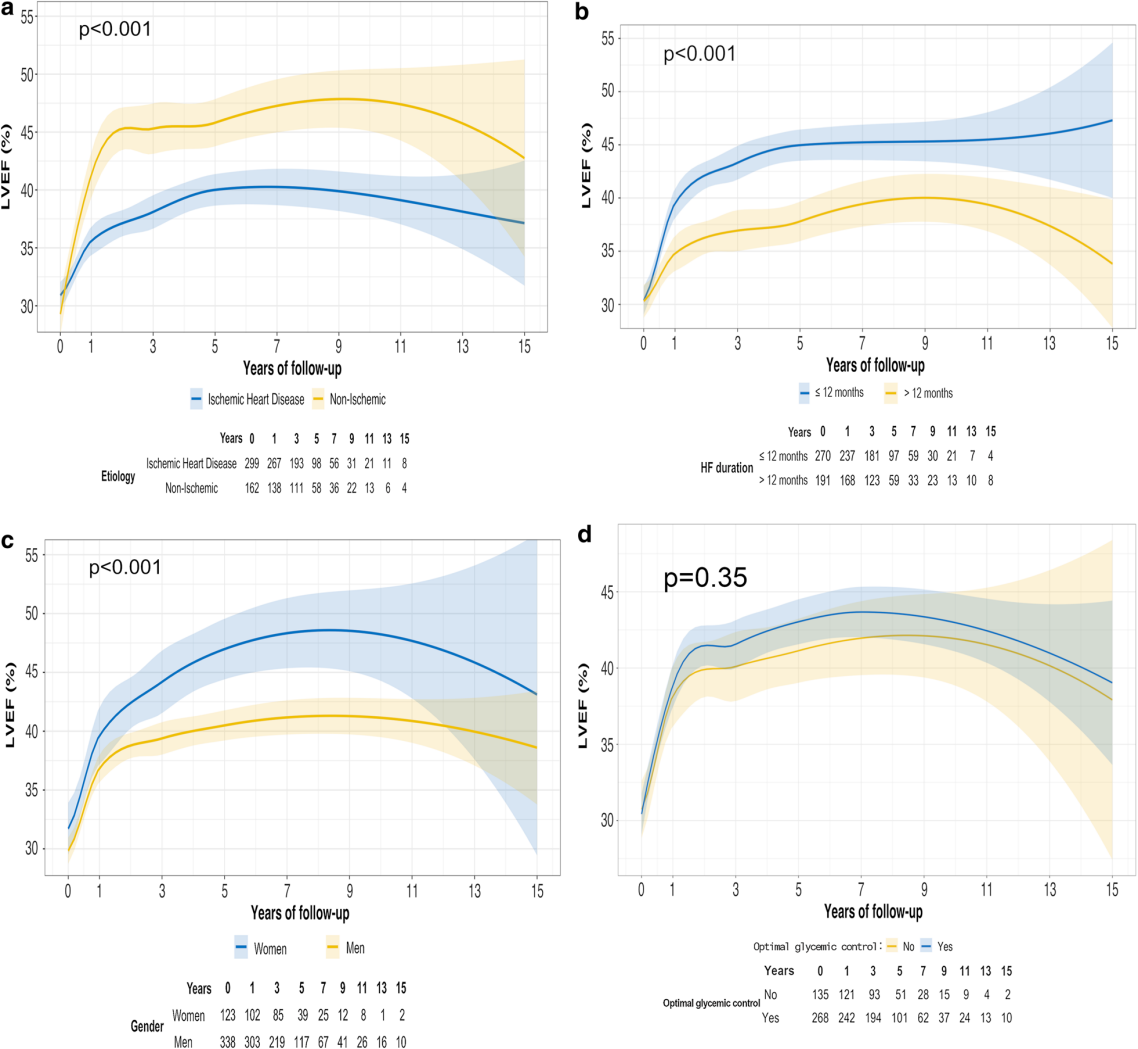
Loess spline curves of long-term LVEF trajectories in diabetic patients based on clinical modifiers. **a** Aetiology: ischemic (blue) vs. non-ischemic (yellow) aetiology. P for trajectory changes on LVEF < 0.001 for both groups. P for comparison between groups < 0.001. Shaded regions displayed around curves represent the confidence interval at level = 0.95. **b** HF Duration: ≤ 12 months (blue) vs. > 12 months (yellow). P value for trajectory changes on LVEF < 0.001 for both groups. P for comparison between groups < 0.001. Shaded regions displayed around curves represent the confidence interval at level = 0.95. **c** Sex: women (blue) vs. men (yellow). P for trajectory changes on LVEF < 0.001 for both sexes. P for comparison between sexes < 0.001. Shaded regions displayed around curves represent the confidence interval at level = 0.95. **d** Optimal (blue) vs. non-optimal glycemic (yellow) control. P for trajectory changes on LVEF < 0.001. P for comparison between glycemic control groups 0.35. Shaded regions displayed around curves represent the confidence interval at level = 0.95

Focusing on diabetic patients (Fig. 3a–c), non-ischemic patients showed a significantly higher LVEF upslope during essentially the entire trajectory (p < 0.001) compared to ischemic patients (Fig. 3a). A more pronounced increase in LVEF during the first year and absence of decrease at the end of follow-up was observed in new-onset HF patients (≤ 12 months) compared to patients with longer HF duration at the baseline visit (Fig. 3b; p < 0.001); and women showed better LVEF improvement than men during nearly all trajectories with a more pronounced inverted U-shape pattern (Fig. 3c; p = 0.03); finally, for those patients with available measurements of HbA1c, no differences in LVEF trajectories were observed based on the optimal/non-optimal glycemic control according to the predefined definition (Fig. 3d; p = 0.32).

Causes of death according to the presence of diabetes are shown in Additional file 2: Table S3. As expected, mortality was higher in diabetic patients, mainly due to HF progression. Cox regression analyses for all-cause death and for the composite end-point of all-cause death or HF-related hospitalization are shown in Additional file 2: Table S4. Furthermore, a worse prognosis was seen in patients with T2D and ischemic heart disease, while non-diabetic patients with non-ischemic etiology showed the best prognosis (Additional file 7: Figure S4A and Additional file 8: Figure S4B). Remarkably, diabetic patients with non-ischemic etiology had a slightly worse prognosis than non-diabetic patients with ischemic etiology. So, the presence of T2D was associated with a higher morbidity and mortality, regardless of whether HF was of ischemic or non-ischemic etiology. Within diabetic patients, in a sensitivity analysis, those with optimal glycemic control showed better prognosis than those with non-optimal control (HR 0.75 [0.55–1.02], p = 0.07 for all-cause death and HR 0.75 [0.57–0.97], p = 0.03 for the composite end-point respectively, after adjustment by age, sex, baseline LVEF, ischemic etiology and NHYA functional class. Finally, diabetic patients showed a double number of recurrent admissions (23.4 vs. 11.2 per 100 patient-years, p < 0.001). In multivariable negative binomial regression analysis, adjusted IRR was 2.0 (1.15–3.48), p = 0.02. Within diabetic patients we did not find significant relationship between the degree of glycemic control and recurrent HF admissions (IRR 0.69 [0.30–1.58], p = 0.37).

## Discussion

Large epidemiological studies have confirmed that T2D is an independent risk factor for the development of HF [3]. It is known that diabetic patients with HF have a higher all-cause mortality and cardiovascular mortality than non-diabetic HF patients [5, 6]. Moreover, following the first HF hospitalization, the incidence of new-onset diabetes was around 2% per year, rising to 3% after 5 years of follow-up, and it was associated with an increased risk of death in established HF patients [16]. However, little is known about the comparison of LVEF trajectories over time between diabetic and non-diabetic patients. Focusing on diabetic patients, the present study expands the prospective assessment of LVEF trajectories in a consecutive real-life cohort of HF patients with depressed systolic function and echocardiographic studies performed over 15 years [10]. LVEF is a key predictor of survival and decreases over time are associated with reduced survival [17, 18]. The transient nature of LVEF recovery has been described previously in patients with depressed systolic function among T2D patients [17–20]. However, none of these studies compared LVEF trajectories along follow-up between HF diabetic and non-diabetic patients. Cioffi et al. [19], in a small prospective short-term study that included patients with LVEF < 40% (22% had T2D), reported LVEF normalization in 22% of non-diabetic HF patients, whereas in diabetic population the LVEF recovery only occurred in 4% of them. The present is the first to report the trajectory of LVEF over very long-term follow-up in patients with T2D and HF compared to non-diabetic HF patients.

### Differences in long-term LVEF trajectory between HF patients according to T2D status

The main finding of the current study was that LVEF trajectories in diabetic and non-diabetic patients were significantly different, as diabetic patients had a more pronounced inverted U shape of LVEF trajectory. This phenomenon suggests a component of diabetic cardiomyopathy. Although the presence of diabetes-related cardiomyopathy is still controversial, a number of studies in both animals and humans have shown structural and functional changes of the diabetic heart, independent of comorbid conditions such as coronary artery disease, hypertension, or valvular heart disease [21]. It has been proposed that the development of diabetic cardiomyopathy is likely to be multifactorial; several mechanisms that include metabolic disturbances, myocardial fibrosis, small vessel disease, autonomic dysfunction, and insulin resistance have been implicated [8, 21]. Focusing on structural alterations, the most prominent histopathological finding in diabetic patients is fibrosis, which result in anatomic and physiological changes in the myocardium. Abnormalities in myocardial metabolism, such as impaired myocardial glucose uptake, have been described in ischemic diabetic experimental models [21]. The increased turnover of free fatty acids seen in diabetic patients may further impair myocardial glucose utilization and may have other deleterious consequences such as changes in myocardial gene expression resulting in myocyte hypertrophy with impaired contractile function models [21, 22].

On the other hand, it is acknowledged that altered LV geometry may precede the development of T2D. A recent report found that LV concentric geometry determined by echocardiography and the severity of LV concentricity evaluated as relative wall thickness (RWT) were associated with incident diabetes in the general population [23]. Furthermore, long-term metformin exposure is associated with protective effects in terms of the incidence of new-onset symptomatic HFpEF, LV diastolic dysfunction and hypertrophy in patients with T2DM and hypertension, which might be beneficial for the delay of HFpEF progression [24].

### Factors influencing LVEF trajectory in T2D patients

The interaction between LVEF trajectory and diabetes was remarkably centered in HF patients with ischemic etiology. We hypothesized that in ischemic diabetic HF patients, other mechanisms directly or indirectly related to diabetes such as specific diabetic cardiomyopathy may also be implicated. On the other hand, a close link between ischemic heart disease and diabetic myocardiopathy may exist. Coronary artery atherosclerosis, a phenomenon associated with diabetes, is directly related to myocardial ischemia, and consequently, impaired myocardial glucose uptake, increased oxidative stress, and vascular endothelial dysfunction, which may promote the progression of diabetic cardiomyopathy.

LVEF trajectories in diabetic patients are also influenced by HF duration and sex. Regarding gender, better LVEF dynamics were observed in women compared to men, regardless of the presence or absence of diabetes. Men are at higher risk of developing left ventricular systolic dysfunction. The exact underlying mechanism is unclear, but sex-related differences in cardiac remodeling and the protective effects of estrogen against apoptosis may be among the explanations. In addition, we found an interaction between diabetes and LVEF trajectory in males; men with diabetes experienced a more pronounced and earlier LVEF decline than those without diabetes. On the contrary we did not find any relationship between glycemic control and LVEF trajectory. There have been conflicting reports regarding the importance of glycemic control in patients with T2D and HF. Our results strongly suggest that other mechanisms rather than on diabetic status also may explain the differences in long-term LVEF trajectory. We must take into account the fact that comorbidities commonly seen in patients with diabetes, such as hypertension, dyslipidemia, and renal impairment, may accelerate the progression of cardiac dysfunction towards advanced disease. In point of fact, the diabetic HF patients in our study had a higher percentage of hypertension and renal insufficiency than the non-diabetics.

Even considering a possible “survival effect,” very long-term survival was accompanied by a progressive LVEF decline, which seemed to be earlier and more pronounced in diabetic patients. We usually continue neurohormonal blockade treatment in all patients irrespective of LVEF increase, as our data support that LVEF improvement represents myocardial remission rather than true myocardial cure, and this issue might be especially important in diabetic patients. This might have important clinical implications, and further research is needed for a better understanding. Yet, a clear direction is lacking on what to do in patients who experience a decline in LVEF over time despite optimal medical treatment.

### Outcomes in patients with and without T2D

Survival and event-free survival curves demonstrated that patients with T2D and ischemic heart disease exhibited the greatest morbidity and mortality after 15 years of follow-up. However, the presence of diabetes was associated with higher events, regardless of whether HF was of ischemic or non-ischemic origin. Some studies have already described that the prognostic impact of diabetes in patients with HF is markedly influenced by the underlying etiology and is particularly deleterious in ischemic heart disease models [4, 25–28]. In a large study published by Johansson et al. [28] that included 35.163 HF patients (24% had T2D), those with diabetes and ischemic heart disease had the highest mortality. Remarkably, in any of these studies, LVEF trajectory has been evaluated.

### Future considerations

Finally, one important question to address in the future is how sodium glucose co-transporter 2 (SGLT2) inhibitors affect LVEF trajectories in T2D with HF. SGLT2 inhibitors act in renal glucose metabolism by inhibiting glucose reabsorption and inducing glycosuria, thereby reducing plasma glucose, blood pressure, and body weight [29]. Several studies in animal models have demonstrated their beneficial effects in left ventricular remodeling, cardiac fibrosis, and vascular dysfunction [30, 31]. Four large cardiovascular outcomes trials with SGLT2 inhibitors have shown important benefits in reducing HF hospitalization by about 30%, even in patients without T2D [32–35]. Several possible mechanisms have been postulated to explain these benefits of SGLT2 inhibitors in HF, but they have not been confirmed [36]. We recently used artificial intelligence to describe that empagliflozin interacts and blocks the sodium-hydrogen exchanger co-transporter and might ameliorate cardiomyocyte cell death [37]. In our cohort, none of the patients were treated with SGLT2 inhibitors, so the results were not influenced by the possible effects of these drugs. On the other hand, in the Prove-HF trial [38], treatment with sacubitril-valsartan was associated with a significant improvement in LVEF during the first year of treatment. In our cohort only 3.8% received such treatment and none of them during the first year of follow-up, so whether this treatment might influence LVEF long-term trajectory is not elucidated yet.

### Study limitations

This study had some limitations. Patients were classified into non-diabetic and diabetic subgroups according to their baseline diagnosis and no data on new-onset T2D diagnosis during follow-up were considered. We don’t have complete data of glycemic control of the whole period of the study and the whole cohort. HbA1c began to be routinely assessed around 2006. LVEF was assessed by transthoracic echocardiography in routine clinical care. However, in the current registry, all echocardiograms were scheduled prospectively and at pre-specified intervals and not at the discretion of the patient’s physician, and were not retrospectively analyzed. We acknowledge that intra- and inter-observer variability of echo-derived LVEF is ~ 5%. However, taking into account the large number of studies performed, we may assume that such variability was randomly distributed during follow-up. On the other hand, contrast echocardiography, longitudinal and radial strain analyses, 3-D echocardiography and cardiac magnetic resonance imaging would evaluate left ventricular function and volumes more precisely and may be superior in the evaluation of LV remodeling parameters, but they are not broadly used in clinical practice. As in all reported studies of variations in LVEF during follow-up, our analyses were performed in “completers,” that is, patients with both baseline and at least other echocardiography study available for analysis. We cannot fully exclude some bias in Loess spline curves due to dropout, as we could not statistically distinguish between autonomous time trends and pseudo upward trends because of successive drop out of fatalities with lower initial LVEF values. Missing data because of loss to follow-up was assumed to be random. Furthermore, Loess spline curve estimations at the end of follow-up were less robust due to the limited number of patients. The study cohort was a general HF population treated at a specific multidisciplinary HF clinic in a tertiary care hospital, with most patients referred from the cardiology department; thus, there was a predominance of relatively young men with HF of ischemic etiology, and an almost exclusively white population, so it may not be possible to fully extrapolate the findings to other populations. Of note, a common treatment protocol was applied to all patients, thereby limiting possible bias introduced by different management strategies or treatment protocols.

To provide more insight into understanding of the mechanisms involved in the “U-effect curve” observed in T2D patients, experimental models might be useful. These studies might contribute to assess the effect of hyperglycemia and insulin-resistance in the pathogenesis of diabetic cardiomyopathy.

## Conclusions

LVEF in T2D with depressed systolic function showed an early improvement with a pronounced inverted U shape of trajectory and marked LVEF decline in the long term. This interaction was mainly observed in patients of ischemic etiology, in whom a component of diabetic cardiomyopathy might be present. In diabetic patients, LVEF trajectories varied upon disease modifiers such as HF etiology, HF duration, and sex.

## Supplementary information

**Additional file 1: Figure S1.** Distribution of the number of echocardiograms performed per patient. Number of echocardiograms per patient ranged from 2 (minimum inclusion criteria) to 9 (patients with all the per protocol pre-specified echocardiograms).

**Additional file 2: Table S1.** Demographic, clinical, and therapeutic characteristics at baseline and treatments during follow-up according to etiology of heart failure and presence of diabetes mellitus. **Table S2.** Paired wise means data analysis in diabetic patients. **Table S3.** Causes of death of the studied cohort during the 15-year follow-up, according the presence or absence of diabetes mellitus. **Table S4.** Multivariable Cox regression analysis for all-cause death and the composite end-point all-cause death or heart failure hospitalization.

**Additional file 3: Figure S2.** Loess spline curves of long-term LVEF trajectories based on heart failure duration. **Panel A**: Patients with HF duration ≤ 12 months; Diabetic (orange) vs. non-diabetic (blue) patients. P value for trajectory changes on LVEF < 0.001 for both groups. P for comparison between groups (interaction between trajectory changes and diabetes) = 0.22. Shaded regions displayed around curves represent the confidence interval at level = 0.95.

**Additional file 4: Figure S2.** Loess spline curves of long-term LVEF trajectories based on heart failure duration. **Panel B**: Patients with HF duration > 12 months. P value for trajectory changes on LVEF < 0.001 for both groups. P for comparison between groups (interaction between time and diabetes) = 0.008. Shaded regions displayed around curves represent the confidence interval at level = 0.95.

**Additional file 5: Figure S3.** Loess spline curves of long-term LVEF trajectories based on sex. **Panel A**: Men. Diabetic (orange) vs. non-diabetic (blue) patients. P value for trajectory changes on LVEF < 0.001 for both groups. P for comparison between groups (interaction between trajectory changes and diabetes) = 0.04. Shaded regions displayed around curves represent the confidence interval at level = 0.95.

**Additional file 6: Figure S3.** Loess spline curves of long-term LVEF trajectories based on sex. **Panel B**: Women. P value for trajectory changes on LVEF <0.001 for both groups. P for comparison between groups (interaction between trajectory changes and diabetes) = 0.14. Shaded regions displayed around curves represent the confidence interval at level = 0.95.

**Additional file 7: Figure S4.** Survival and event-free survival curves related to the presence of diabetes mellitus and to etiology (ischemic vs. non-ischemic). **Panel A:** All-cause death survival curves.

**Additional file 8: Figure S4.** Survival and event-free survival curves related to the presence of diabetes mellitus and to etiology (ischemic vs. non-ischemic). **Panel B:** Event-free survival curves (composite end-point of all-cause death or HF hospitalizations). Diabetic patients from ischemic etiology (dark purple) showed the worse prognosis, while non-diabetic from non-ischemic etiology (blue) showed the best. Remarkably diabetic patients from non-ischemic etiology (soft orange) showed slightly worse prognosis than non-diabetic patients from ischemic etiology (green).

## Data Availability

The datasets used and/or analysed during the current study are available from the corresponding author on reasonable request.

## Abbreviations

HF: Heart failure
LME: Linear Mixed-Effects
Loess: Locally weighted error sum of squares
LVEF: Left ventricular ejection fraction
T2D: Type 2 diabetes
