# Supplementary figures and images for: Long-term LVEF trajectories in patients with type 2 diabetes and heart failure: diabetic cardiomyopathy may underlie functional decline
Source: Cardiovasc Diabetol. 2020 Mar 23;19:38. doi: 10.1186/s12933-020-01011-w (PMC7092450; doi:10.1186/s12933-020-01011-w)

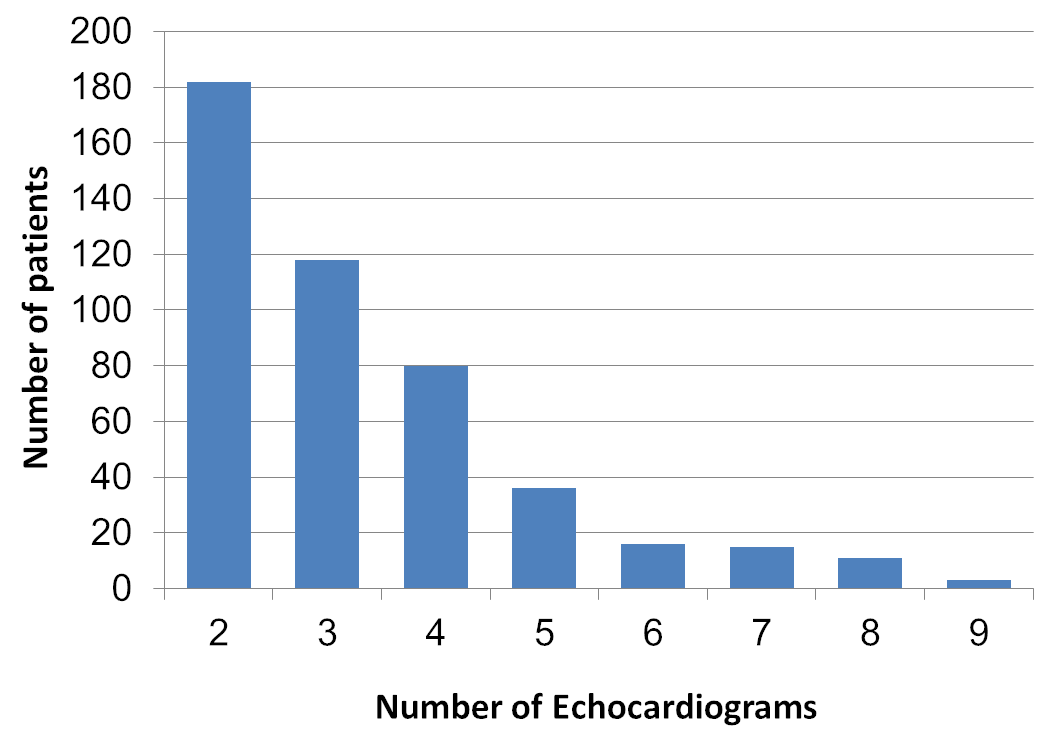

Supplement: Supplementary file 1 — Additional file 1: Figure S1. Distribution of the number of echocardiograms performed per patient. Number of echocardiograms per patient ranged from 2 (minimum inclusion criteria) to 9 (patients with all the per protocol pre-specified echocardiograms). [file 12933_2020_1011_MOESM1_ESM.tif]

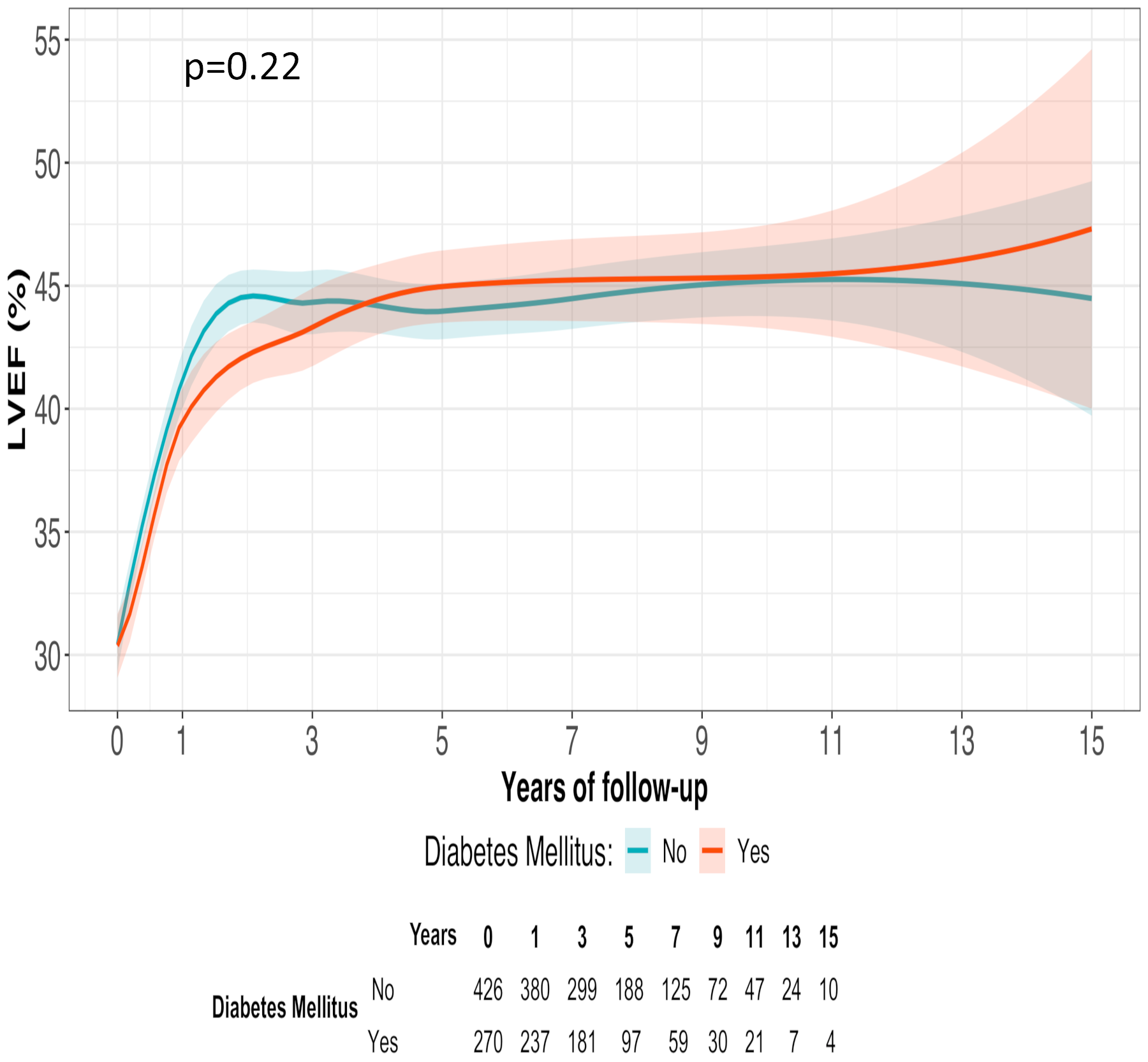

Supplement: Supplementary file 3 — Additional file 3: Figure S2. Loess spline curves of long-term LVEF trajectories based on heart failure duration. Panel A: Patients with HF duration ≤ 12 months; Diabetic (orange) vs. non-diabetic (blue) patients. P value for trajectory changes on LVEF < 0.001 for both groups. P for comparison between groups (interaction between trajectory changes and diabetes) = 0.22. Shaded regions displayed around curves represent the confidence interval at level = 0.95. [file 12933_2020_1011_MOESM3_ESM.tif]

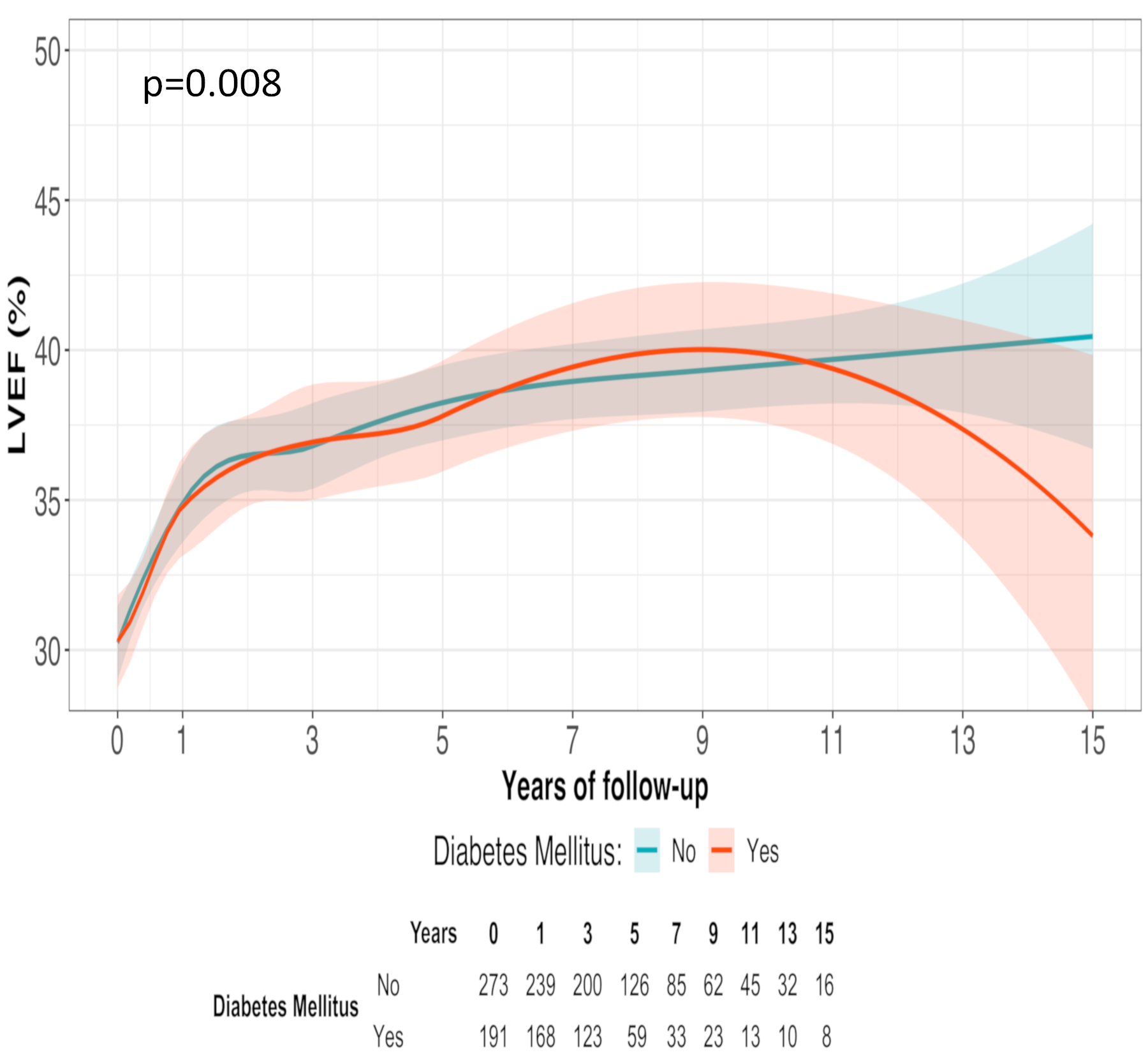

Supplement: Supplementary file 4 — Additional file 4: Figure S2. Loess spline curves of long-term LVEF trajectories based on heart failure duration. Panel B: Patients with HF duration > 12 months. P value for trajectory changes on LVEF < 0.001 for both groups. P for comparison between groups (interaction between time and diabetes) = 0.008. Shaded regions displayed around curves represent the confidence interval at level = 0.95. [file 12933_2020_1011_MOESM4_ESM.tif]

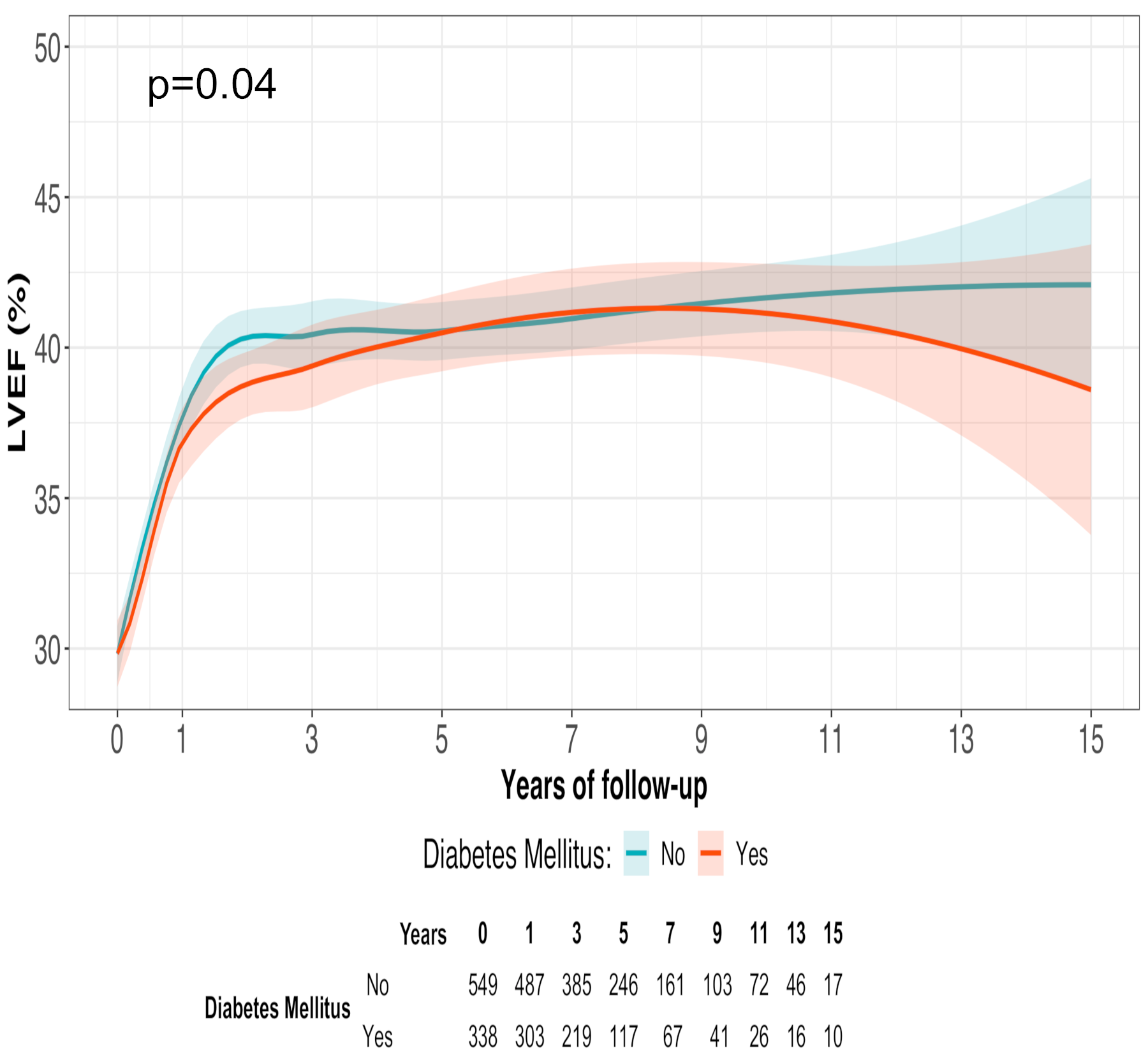

Supplement: Supplementary file 5 — Additional file 5: Figure S3. Loess spline curves of long-term LVEF trajectories based on sex. Panel A: Men. Diabetic (orange) vs. non-diabetic (blue) patients. P value for trajectory changes on LVEF < 0.001 for both groups. P for comparison between groups (interaction between trajectory changes and diabetes) = 0.04. Shaded regions displayed around curves represent the confidence interval at level = 0.95. [file 12933_2020_1011_MOESM5_ESM.tif]

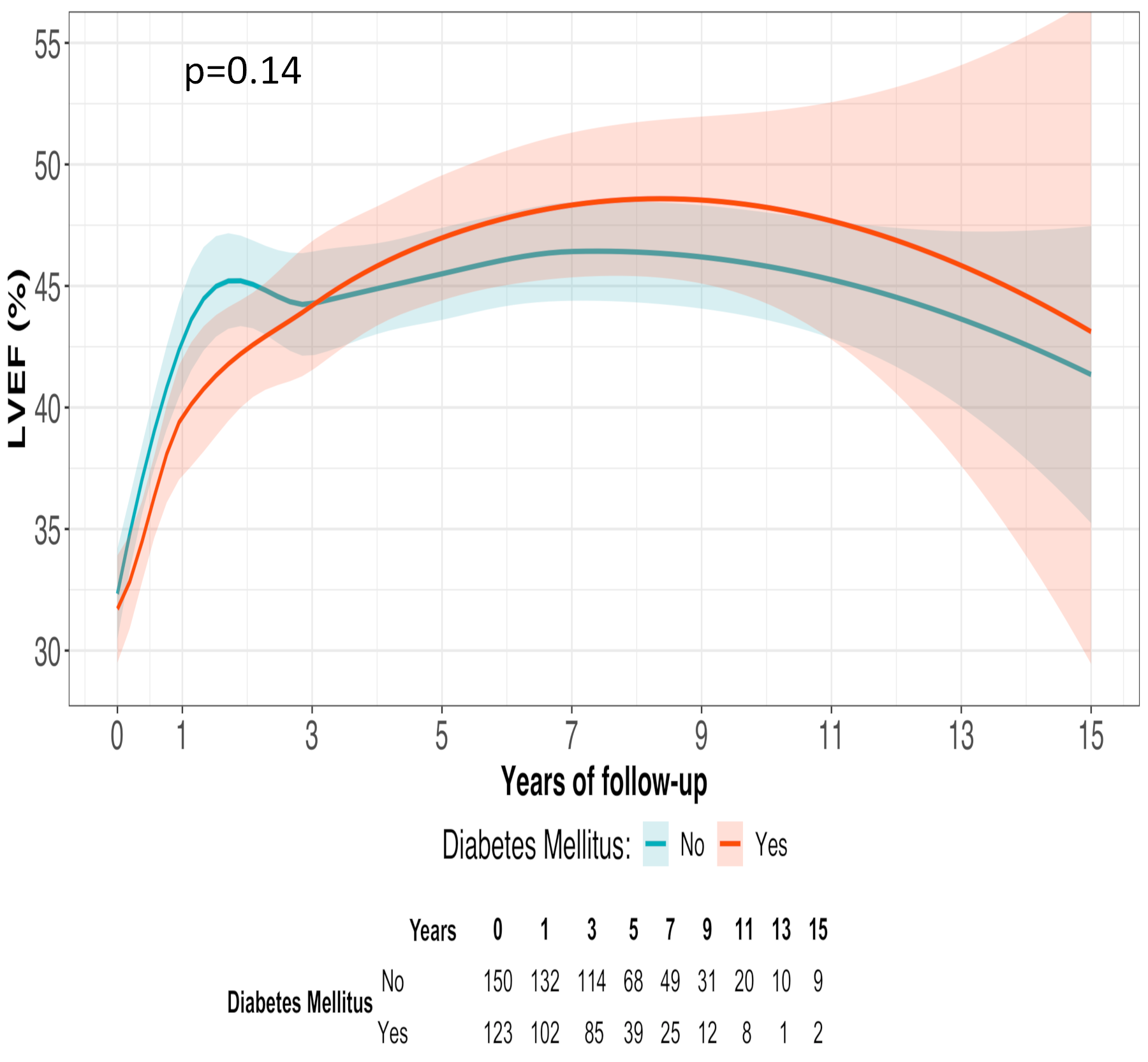

Supplement: Supplementary file 6 — Additional file 6: Figure S3. Loess spline curves of long-term LVEF trajectories based on sex. Panel B: Women. P value for trajectory changes on LVEF <0.001 for both groups. P for comparison between groups (interaction between trajectory changes and diabetes) = 0.14. Shaded regions displayed around curves represent the confidence interval at level = 0.95. [file 12933_2020_1011_MOESM6_ESM.tif]

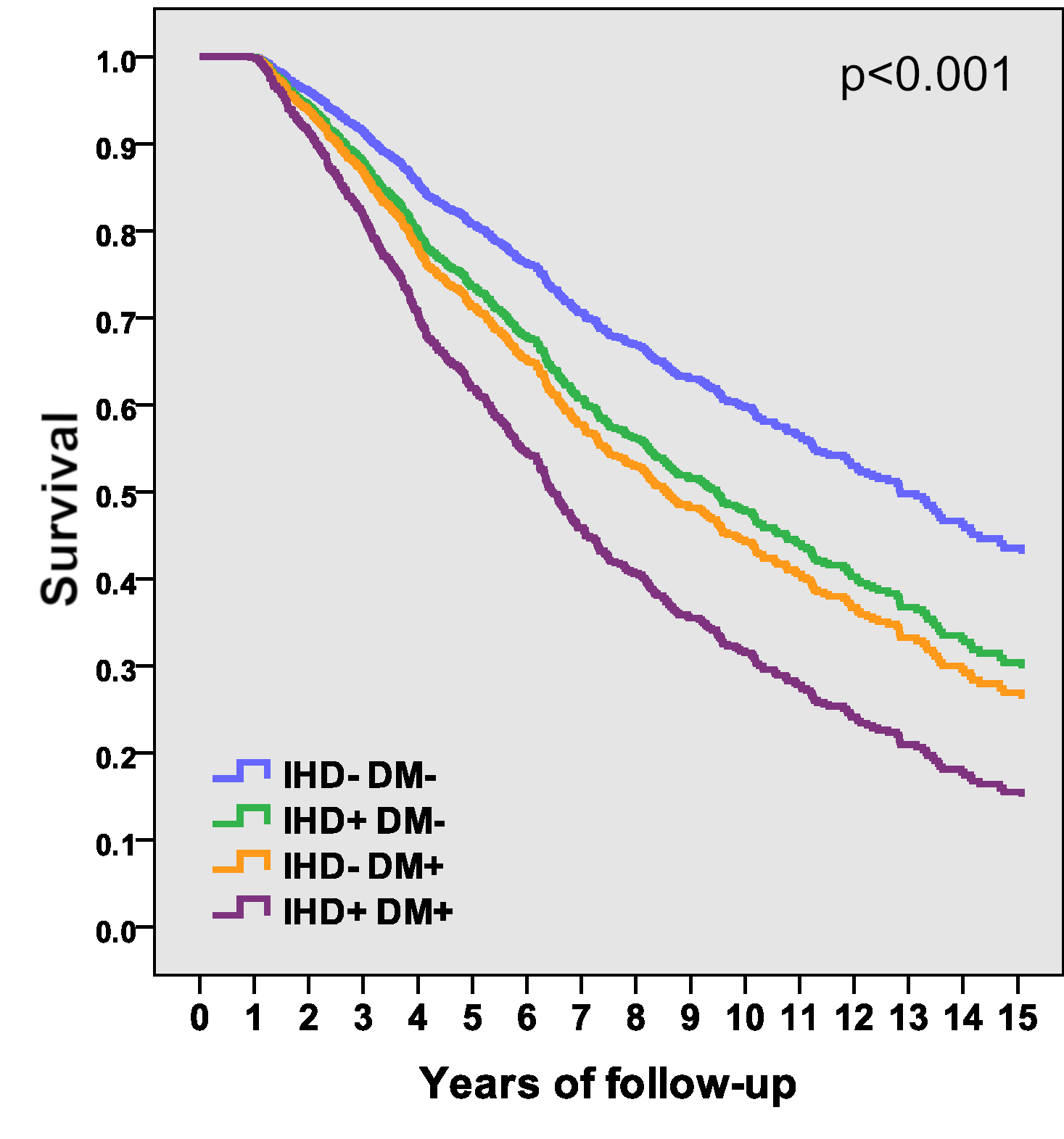

Supplement: Supplementary file 7 — Additional file 7: Figure S4. Survival and event-free survival curves related to the presence of diabetes mellitus and to etiology (ischemic vs. non-ischemic). Panel A: All-cause death survival curves. [file 12933_2020_1011_MOESM7_ESM.tif]

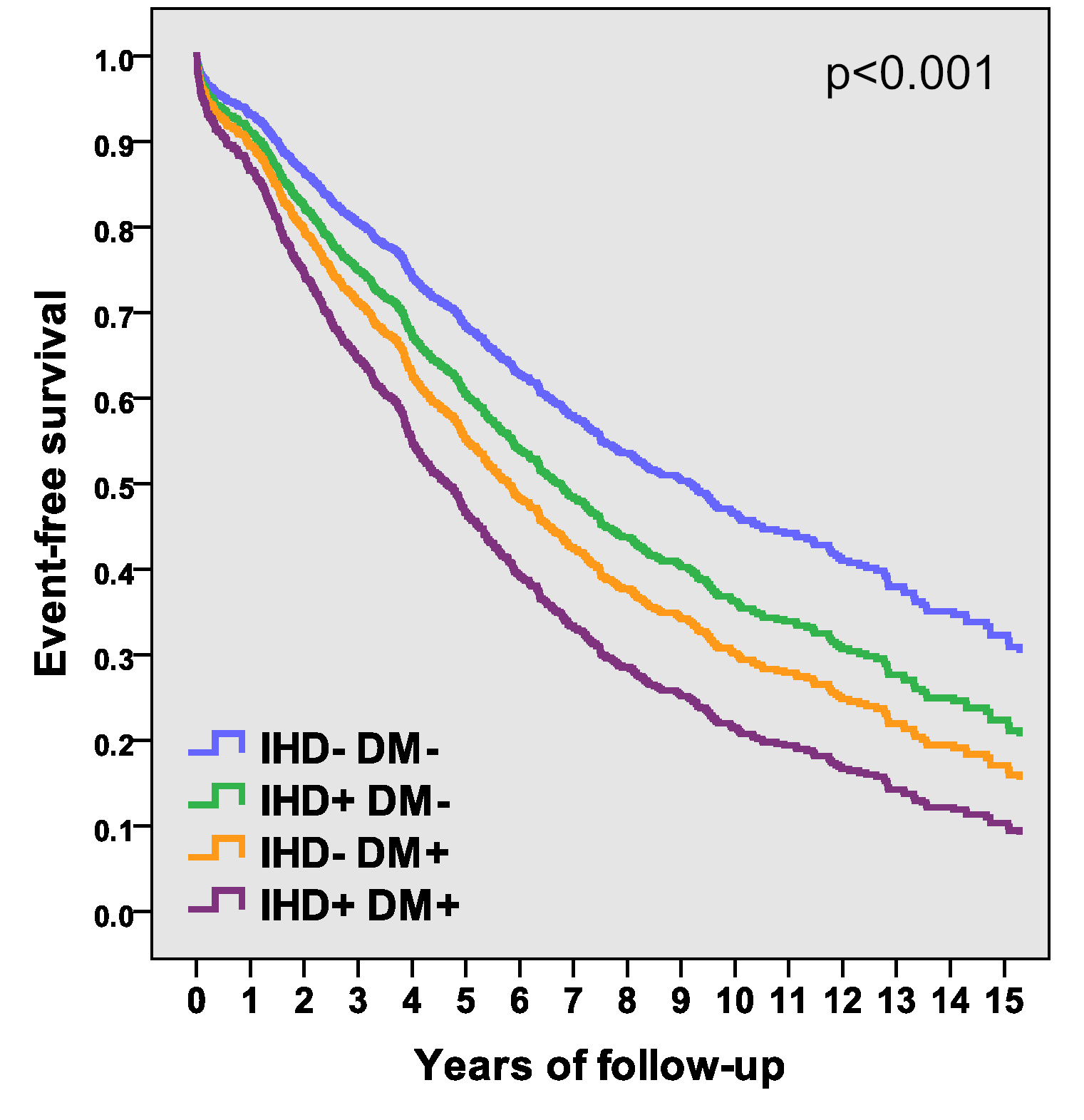

Supplement: Supplementary file 8 — Additional file 8: Figure S4. Survival and event-free survival curves related to the presence of diabetes mellitus and to etiology (ischemic vs. non-ischemic). Panel B: Event-free survival curves (composite end-point of all-cause death or HF hospitalizations). Diabetic patients from ischemic etiology (dark purple) showed the worse prognosis, while non-diabetic from non-ischemic etiology (blue) showed the best. Remarkably diabetic patients from non-ischemic etiology (soft orange) showed slightly worse prognosis than non-diabetic patients from ischemic etiology (green). [file 12933_2020_1011_MOESM8_ESM.tif]
