# Supplementary material for: Long-term LVEF trajectories in patients with type 2 diabetes and heart failure: diabetic cardiomyopathy may underlie functional decline
Source: Cardiovasc Diabetol. 2020 Mar 23;19:38. doi: 10.1186/s12933-020-01011-w (PMC7092450; doi:10.1186/s12933-020-01011-w)
Supplement: Supplementary file 2 — Additional file 2: Table S1. Demographic, clinical, and therapeutic characteristics at baseline and treatments during follow-up according to etiology of heart failure and presence of diabetes mellitus. Table S2. Paired wise means data analysis in diabetic patients. Table S3. Causes of death of the studied cohort during the 15-year follow-up, according the presence or absence of diabetes mellitus. Table S4. Multivariable Cox regression analysis for all-cause death and the composite end-point all-cause death or heart failure hospitalization. [file 12933_2020_1011_MOESM2_ESM.docx]

**Table S1. Demographic, clinical, and therapeutic characteristics at baseline and treatments during follow-up according to etiology of heart failure and presence of diabetes mellitus**

|  | **ISCHEMIC ETIOLOGY** | | | **NON-ISCHEMIC ETIOLOGY** | | |
| --- | --- | --- | --- | --- | --- | --- |
|  | **Diabetics** | **Non-Diabetics** | **p-value** | **Diabetics** | **Non-Diabetics** | **p-value** |
|  | **N=298** | **N=361** |  | **N=163** | **N=338** |  |
| Age, years | 66.9 ± 10.1 | 66.2 ± 11.9 | 0.37 | 65.3 ± 11.1 | 61.6 ± 14.2 | 0.004 |
| Male | 226 (75.8) | 303 (83.9) | 0.009 | 112 (68.7) | 246 (72.8) | 0.35 |
| White | 297 (99.7) | 359 (99.4) | 0.68 | 162 (99.4) | 332 (92.8) | 0.30 |
| HF duration, months | 7 (2–40) | 6 (1–40) | 0.19 | 7 (1.52–38) | 4.5 (1–41) | 0.32 |
| NYHA class |  |  | 0.06 |  |  | 0.03 |
| I | 10 (3.4) | 21 (5.8) |  | 4 (2.5) | 25 (7.4) |  |
| II | 204 (68.5) | 258 (71.5) |  | 114 (69.9) | 241 (71.3) |  |
| III | 81 (27.2) | 82 (22.7) |  | 44 (27.0) | 70 (20.7) |  |
| IV | 3 (1.0) | 0 (0) |  | 1 (0.6) | 2 (0.6) |  |
| LVEF, % | 30.9 ± 8.2 | 30.9 ± 7.9 | 1.00 | 29.3 ± 8.6 | 29.7 ± 9.2 | 0.67 |
| LVEDD, mm | 60 ± 7.8 | 61 ± 7.8 | 0.13 | 61.8 ± 7.9 | 62.8 ± 9.3 | 0.29 |
| LVESD, mm | 47.5 ± 9.3 | 48.4 ± 9.2 | 0.20 | 50.2 ± 9.2 | 51.3 ± 9.8 | 0.27 |
| Hypertension | 214 (71.8) | 205 (56.8) | <0.001 | 119 (73.0) | 175 (51.8) | <0.001 |
| Anemia* | 166 (57.6) | 158 (45.1) | 0.002 | 66 (42.3) | 90 (27.0) | 0.001 |
| Renal insufficiency† | 156 (52.7) | 136 (38.2) | <0.001 | 83 (51.2) | 113 (33.8) | <0.001 |
| Atrial fibrillation/flutter | 34 (11.4) | 41 (11.4) | 0.98 | 46 (28.2) | 82 (24.3) | 0.34 |
| LBBB | 33 (11.1) | 37 (10.2) | 0.73 | 27 (16.6) | 58 (17.2) | 0.87 |
| Heart rate, bpm | 71.1 ± 12.9 | 67.8 ± 13.9 | 0.002 | 74.3 ± 14.5 | 71.6 ± 16.0 | 0.06 |
| Blood pressure, mmHg | 126.5 ± 22.6 | 121.5 ± 20.3 | 0.003 | 126.5 ± 20.5 | 128.3 ± 22.4 | 0.38 |
| BMI, Kg/m2 | 27.6 (25-30.9) | 26.6 (24.1-29.7) | 0.003 | 27.9 (25-32) | 26.4 (23.7-29.7) | <0.001 |
| NTproBNP, ng/L | 1896 (884–4578) | 1900 (780–4016) | 0.40 | 1560 (720–3824) | 1310 (497–2937) | 0.04 |
| HF Treatments (F-U), n (%) |  |  |  |  |  |  |
| ACEI or ARB | 266 (89.3) | 343 (95.0) | 0.006 | 155 (95.1) | 320 (94.7) | 0.84 |
| Beta-blocker | 285 (95.6) | 346 (95.8) | 0.90 | 151 (92.6) | 312 (92.3) | 0.90 |
| MRA | 203 (68.1) | 221 (61.2) | 0.07 | 130 (79.8) | 224 (66.3) | 0.002 |
| Loop diuretic | 286 (96) | 312 (86.4) | <0.001 | 155 (95.1) | 309 (91.4) | 0.14 |
| Digoxin | 118 (39.6) | 109 (30.2) | 0.01 | 95 (58.3) | 155 (45.9) | 0.009 |
| Ivabradine | 66 (22.1) | 53 (14.7) | 0.01 | 47 (28.8) | 69 (20.4) | 0.04 |
| Sacubitril/Valsartan | 11 (3.7) | 17 (4.7) | 0.52 | 3 (1.8) | 13 (3.8) | 0.23 |
| CRT | 9 (3.0) | 22 (6.1) | 0.06 | 14 (8.6) | 23 (6.8) | 0.45 |
| ICD | 45 (15.1) | 80 (22.2) | 0.02 | 17 (10.4) | 29 (8.6) | 0.50 |
| Antidiabetic treatments |  |  |  |  |  |  |
| Oral drugs baseline | 158 (53) |  |  | 90 (55.2) |  |  |
| Insulin baseline | 130 (43.6) |  |  | 45 (27.6) |  |  |
| Oral drugs F-U | 231 (77.5) |  |  | 136 (83.4) |  |  |
| Insulin F-U | 202 (67.8) |  |  | 103 (63.2) |  |  |
|  |  |  |  |  |  |  |

Data in mean ± SD, median (IQR) or n (%)

*According to W.H.O. criteria (<13 g/dl in men and <12 g/dl in women)

†eGFR (CKD-EPI equation) < 60 ml/min/1.73 m^2^

ACEI: angiotensin converting enzyme inhibitor; ARB: angiotensin II receptor blocker; BMI: body mass index; CRT: cardiac resynchronization therapy; eGRF: estimated glomerular renal filtration (CKD-EPI equation); F-U: follow-up; HF: heart failure; ICD: implantable cardiac defibrillator; LBBB: left bundle branch block. LVEF: left ventricular ejection fraction; LVEDD: left ventricular end-diastolic diameter; LVESD: left ventricular end-systolic diameter; MRA: mineralcorticoid recptor antagonist; NYHA: New York Heart Association; NTproBNP: N-terminal pro-brain natriuretic peptide.

**Table S2.** Paired wise means data analysis in diabetic patients

|  |  | **LVEF** | **Mean change** | **p-value** | **N** |
| --- | --- | --- | --- | --- | --- |
| Pair 1 | Baseline | 30.5 ± 8.3 |  |  | 417 |
|  | 1 year | 37.9 ± 11.6 | +7.4 ± 11.4 | <0.001 |  |
| Pair 2 | 1 year | 38.6 ± 11.4 |  |  | 266 |
|  | 3 years | 40.6 ± 11.8 | +2 ± 8.4 | <0.001 |  |
| Pair 3 | 3 years | 42 ± 12.1 |  |  | 147 |
|  | 5 years | 42.5 ± 12.1 | +0.46 ± 8 | 0.48 |  |
| Pair 4 | 5 years | 42.4 ± 12.4 |  |  | 79 |
|  | 7 years | 44.1 ±13.3 | +1.7 ± 7.7 | 0.051 |  |
| Pair 5 | 7 years | 45.2 ± 13.6 |  |  | 42 |
|  | 9 years | 43.3 ± 12.2 | -1.94 ± 9.6 | 0.20 |  |
| Pair 6 | 9 years | 41.8 ± 12.7 |  |  | 28 |
|  | 11 years | 41.2 ± 11.1 | -0.6 ± 9.2 | 0.73 |  |
| Pair 7 | 11 years | 40.4 ± 9.3 |  |  | 13 |
|  | 13 years | 39 ± 10.2 | -1.4 ± 4.6 | 0.30 |  |
| Pair 8 | 13 years | 40 ± 10.6 |  |  | 11 |
|  | 15 years | 39.8 ± 11.9 | -0.3 ± 4.4 | 0.21 |  |

**Table S3**. Causes of death of the studied cohort during the 15-year follow-up, according the presence or absence of diabetes mellitus.

|  |  |  |  |
| --- | --- | --- | --- |
|  | **Diabetics** | **Non-diabetics** |  |
|  | **N=461** | **N=699** | **p-value** |
| **Cause of death, n (%)** |  |  |  |
| Unknown | 21 (4.5) | 16 (2.3) | 0.03 |
| Heart failure | 80 (17.4) | 66 (9.4) | <0.001 |
| Sudden death | 37 (8.0) | 41 (5.9) | 0.15 |
| AMI | 13 (2.8) | 12 (1.7) | 0.21 |
| Stroke | 2 (0.4) | 5 (0.7) | 0.55 |
| CV procedure | 3 (0.7) | 5 (0.7) | 0.90 |
| Non-CV | 78 (16.9) | 124 (17.7) | 0.72 |
| Other CV | 11 (2.9) | 22 (3.1) | 0.45 |
|  |  |  |  |
| **Total dead** | 245 (53.1) | 291 (41.6) | <0.001 |

AMI: acute myocardial infarction; CV: cardiovascular

**Table S4.** Multivariable Cox regression analysis for all-cause death and the composite end-point all-cause death or heart failure hospitalization.

|  | **All-cause death** | | | **Composite end-point** | | |
| --- | --- | --- | --- | --- | --- | --- |
|  | **HR** | **95%CI** | **p-value** | **HR** | **95%CI** | **p-value** |
| Age | 1.06 | 1.05-1,07 | <0.001 | 1.04 | 1.03-1.05 | <0.001 |
| Female sex | 0.73 | 0.59-0.90 | 0.003 | 0.91 | 0.75-1.09 | 0.30 |
| Ischemic etiology | 1.28 | 1.06-1.53 | 0.009 | 1.24 | 1.05-1.45 | 0.01 |
| NYHA functional class | 1.55 | 1.32-1.81 | <0.001 | 1.39 | 1.20-1.60 | <0.001 |
| LVEF | 0.99 | 0.98-1.00 | 0.02 | 0.99 | 0.98-1.00 | 0.02 |
| Diabetes | 1.54 | 1.30-1.83 | <0.001 | 1.46 | 1.25-1.70 | <0.001 |

NYHA: New York Heart Association; LVEF: left ventricular ejection fraction
